# In Vitro Effects of Hollow Gold Nanoshells on Human Aortic Endothelial Cells

**DOI:** 10.1186/s11671-016-1620-5

**Published:** 2016-09-13

**Authors:** Chunrong Gu, Hengfang Wu, Gaoyuan Ge, Xiongzhi Li, Zhirui Guo, Zhiping Bian, Jindan Xu, Hua Lu, Xiangjian Chen, Di Yang

**Affiliations:** 1 Research Institute of Cardiovascular Disease, The First Affiliated Hospital of Nanjing Medical University, 300 Guangzhou Road, Nanjing, 210029 China; 2 Department of Cardiology, The First Affiliated Hospital of Nanjing Medical University, 300 Guangzhou Road, Nanjing, 210029 China; 3 The Second Affiliated Hospital of Nanjing Medical University, Nanjing, 210011 China; 4 State Key Laboratory of Bioelectronics, School of Biological Science and Medical Engineering, Southeast University, Nanjing, 210096 China

**Keywords:** Hollow gold nanoshells, Human aortic endothelial cells, Cell viability, Angiogenesis

## Abstract

Gold nanoparticles are emerging as promising biomedical tools due to their unique nanoscale characteristics. Our purpose was to synthesize a hollow-shaped gold nanoparticle and to investigate its effect on human aortic endothelial cells (HAECs) in vitro. Hollow gold nanoshells with average 35-nm diameters and 10-nm shell thickness were obtained by galvanic replacement using quasi-spherical nanosilver as sacrifice-template. Our results showed that hollow gold nanoshells in the culture medium could be internalized into the cytoplasm of HAECs. No cytotoxicity effect of hollow gold nanoshells on HAECs was observed within the test concentrations (0–0.8 μg/mL) and test exposure period (0–72 h) by tetrazolium dye assay. Meanwhile, the release of cell injury biomarker, lactate dehydrogenase, was not significantly higher than that from control cells (without hollow gold nanoshells). The concentrations of vasodilators, nitric oxide, and prostacyclin I-2 were not changed, but the vasoconstrictor endothelin-1 was decreased by hollow gold nanoshells treatment in HAECs. HAECs exposed to hollow gold nanoshells resulted in suppressing expressions of genes involved in apoptosis and activating expressions of genes of adhesion molecules. Moreover, we demonstrated by in vitro endothelial tube formation that hollow gold nanoshells (0.8 μg/mL) could not inhibit angiogenesis by the HAECs. Altogether, these results indicate that the structure and major function of HAECs would not be disrupted by hollow gold nanoshell treatment.

**Electronic supplementary material:**

The online version of this article (doi:10.1186/s11671-016-1620-5) contains supplementary material, which is available to authorized users.

## Background

Metal nanoparticles are made of elemental metals and their oxides and compounds, such as iron, silicon, zinc, silver, and notably gold nanoparticles [1]. Gold nanoparticles have been applied for diagnostic imaging, vaccines, biosensing, cancer therapy, and drug delivery. The engineered gold nanoparticles can be well controlled for appropriated usage. Different forms of gold nanoparticles, such as gold nanospheres, gold nanorods, silica/gold nanoshells, and hollow gold nanospheres, have been synthesized with special optical and electric properties [2].

With the studies of gold nanoparticles synthesis, modification, and application, focus is given on the interactions between the nanoparticles and the cells. The unique, tunable, and versatile physicochemical properties of nanoparticles, such as the size, shape, and surface modifications, directly influence the nano-bio interaction, including cellular uptake, intracellular trafficking, and toxic response [3]. Once inside the human body via the skin, lung, and digestive system, the nanoparticles often penetrate the barriers, enter the bloodstream, and directly interact with the vascular endothelial cells (ECs). Vascular ECs form the inner lining of all blood vessels and possess vital synthetic, secretory, metabolic, and immunological functions. The arterial and venous systems of the cardiovasculature function differently. So, it is not surprising that the arterial and venous endothelial cells are also distinct. Previous studies have shown that the specification of arterial and venous identity is largely genetically determined [4, 5]. The dysfunction of arterial endothelial cells is a hallmark of many pathologic states including atherosclerosis and diabetes mellitus [6].

Previous studies demonstrated the high impact of the shape, size and surface charge of gold nanoparticles on endothelial cells viability and internalization [7]. The elongated shape of gold nanoparticle rods and positive-charged surface (NH_2_, CyA) leaded to strong reduction in cell viability. The endocytotic pathway is probably a size-dependent process with caveolae-mediated uptake of gold nanoparticles around 20 nm and clathrin- or macropinocytosis-mediated internalization of gold nanoparticles greater than 40 nm [7]. Others demonstrated that the main parameter in the evaluation of the gold nanoparticles toxicity on endothelial cells was the roughness of gold nanoparticles [8], and this effect was independent [8] or dependent [9] on the surface chemistry. Most of the studies employed venous endothelial cells, such as human umbilical vein endothelial cells (HUVECs), to evaluate the biocompatibility of nanoparticles on endothelial cells [10–13]. In this study, we successfully synthesized naked hollow gold nanoshells by galvanic replacement using quasi-spherical silver nanoparticles as sacrifice-template. We assessed the potential cytotoxic effect of the synthesized naked hollow gold nanoshells on human aortic endothelial cells (HAECs), which are cells derived from the endothelium of artery from the human aorta.

## Methods

### Preparation of Hollow Gold Nanoshells

Hollow gold nanoshells were obtained by galvanic replacement using quasi-spherical silver nanoparticles as sacrifice-template. These silver nanoparticles were synthesized by a seed-mediated route previously reported [14]. Briefly, 20 mL of 1 % (*w*/*v*) citrate solution and 75 mL of water were added in a round bottom flask and the mixture was heated to 70 °C and 1.7 mL of 1 % (*w*/*v*) AgNO_3_ solution was introduced to the mixture, followed by adding 2 mL of 0.1 % (w/v) freshly prepared NaBH_4_ solution. The resulting silver nanoparticles (average 4 nm) were used as seeds. Next, 2 mL of 1 % citrate solution was mixed with 80 mL of water in a 250-mL three-necked round bottom flask equipped with a reflux condenser and brought to boiling by a heating mantle. Three milliliters of the seeds solution was added while vigorously mechanical stirring, followed by the addition of 1.7 mL of 1 % AgNO_3_ solution. Stirring continued for 1 h while keeping reflux and cooled to room temperature. Water was added to bring the volume to 100 mL.

Silver nanoparticles synthesized in this way with an average size of 30 nm were used as sacrifice-template. The procedure of fabricating hollow gold nanoshells was as follows: a 30 mL of as-obtained silver nanoparticles was centrifuged at 10,000×*g* per min for 20 min to remove extra citrate or AgNO_3_ and redispersed by deionized water to obtain a 100-mL colloidal solution. This solution was brought to boiling under vigorously mechanical stirring. Next, 2.0 mL of 2 mM HAuCl_4_ was added dropwisely. After that, the reaction solution was kept boiling until no color changes were observed. The resultant solution was then allowed to cool to room temperature under constant stirring.

### Characterization of Hollow Gold Nanoshells

The morphology and structure of the resultants were observed by scanning electron microscopy (SEM). SEM images were obtained with a field emission scanning electron microscope (Carl Zeiss) operated at 20 kV. The samples were prepared by dropping the dispersion of hollow gold nanoshell products onto the piranha-processed silicon wafer and dried in ambience. The average sizes of as-obtained hollow gold nanoshells were measured from about 100 particles to provide statistical significance. The UV-vis extinction spectra were recorded by a Shimadzu UV-3600 spectrophotometer in a range of 300–1100 nm. The zeta potential of the hollow gold nanoshells was measured using a Malvern Zetasizer 3000HSA (Malvern Instruments, Ltd.). The dispersibility of the hollow gold nanoshells in solution was analyzed by a NanoSight LM10-HSBF system (Malvern Instruments Ltd.)

### HAECs Culture

HAECs were used for experiments at passages 2 to 5 in this study to assure the fidelity and consistency of these cells. HAECs were cultured in Dulbecco’s modified Eagle’s medium (DMEM, GIBCO, NY, USA) supplemented with 1 % endothelial cell growth supplement (M&C Gene Technology, Beijing, China), 20 % fetal bovine serum (GIBCO, NY, USA), 1 % heparin sodium, 1 % non-essential amino acid solution (100×, Sigma-Aldrich, MO, USA), 1 % l-glutamine (Sigma-Aldrich, MO, USA), 100 U/mL penicillin, and 100 μg/mL streptomycin (Sigma-Aldrich, MO, USA). The cells were maintained at 37 °C in a humidified incubator with 5 % CO_2_.

### Location of Hollow Gold Nanoshells in the HAEC

The transmission electron microscopy (TEM) analysis was performed to observe whether hollow gold nanoshells were taken up by the HAECs. The HAECs incubated with 0.8 μg/mL hollow gold nanoshells for 24 h were washed with phosphate buffer solution (PBS) and routinely fixed, dehydrated, and embedded. Ultrathin sections (80 nm) were transferred to the 200-mesh copper grid, stained with 5 % lead tetraacetate, air-dried, and then examined with a TEM (JEM-1010) at 80 kV.

### Cytotoxicity Evaluation

The cytotoxicity of hollow gold nanoshells on HAECs was evaluated by the MTT assay (3-(4,5-dimethyl-2-thiazolyl)-2,5-diphenyl-2-H-tetrazolium bromide), which is a simple nonradioactive colorimetric assay to assess cell viability and cytotoxicity. In this study, HAECs were seeded on 96-well plates at a density of 3 × 10^3^ cells per well for 12 h (about 60 % confluent). The hollow gold nanoshells, diluted with culture medium at graded concentrations from 0.008 to 0.8 μg/mL, respectively, were incubated with HAECs for 24 h. In addition, HAECs were incubated with 0.8 μg/mL hollow gold nanoshells for 4, 24, 48, and 72 h, respectively. After washing with PBS, the cells were incubated with 200 μL MTT solution (Amresco, OH, USA, 0.5 mg/mL in DMEM) at 37 °C for 2 h. Then, the cells were washed twice with PBS, and dimethyl sulfoxide (DMSO, Sigma-Aldrich, MO, USA) was added 150 μL per well. The plates were placed for 15 min at room temperature to dissolve the dyes, and then the absorbance was examined at 595 nm by Ultra Microplate Reader ELX808IU (BioTek Instruments, VT, USA) and cell viability was calculated as a percentage of control cells treated without hollow gold nanoshells. Each experiment was repeated at least three times independently.

### HAEC Injury Markers and Vasoregulators

Lactate dehydrogenase (LDH) is a cytoplasmic cellular enzyme, which can be released to extracellular space when the cell membrane is disrupted by pathological conditions. Measuring the LDH concentration in supernatant of cultured HAECs is therefore a good marker for determination of cell membrane integrity [15]. Urea transporters, located in endothelial cells, are responsible for transporting extracellular urea into the cell. Vasodilators (nitric oxide (NO) and prostacyclin I-2 (PGI-2)) and vasoconstrictors (endothelin-1 (ET-1)) can be released by ECs to regulate blood pressure and blood flow. In this study, HAECs were co-cultured with 0.8 μg/mL hollow gold nanoshells for 24 h. Then, the cell culture supernatant was centrifuged at 8000×*g*, 4 °C for 30 min to remove the rest nanoparticles and cell debris. Supernatant LDH and urea were determined using Olympus AU5400 automatic biochemistry analyzer. The concentrations of ET-1, PGI-2, and NO in the supernatant were measured by enzyme-linked immunosorbent assay (ELISA) kits (Jiancheng, Nanjing, China), respectively.

### Real-time PCR Analysis of HAEC Gene Expression

About forty genes related to apoptosis cascade, endoplasmic reticulum (ER) stress, oxidative stress, adhesion molecules, and calcium handling proteins were detected by real-time PCR. In this study, HAECs were incubated with 0.8 μg/mL hollow gold nanoshells for 24 h. Total RNA (300 ng) was reverse transcribed using the PrimeScript^TM^ RT reagent Kit, and then the complementary DNA (cDNA) was amplified using SYBR Premix Ex Taq^TM^ according to the following cycle conditions: 30 s at 95 °C for 1 cycle, 5 s at 95 °C, and 30 s at 60 °C for 40 cycles (AB 7900HT Fast Real-Time PCR system). All real-time PCR reactions were performed in triplicate. The housekeeping gene GAPDH was used as an internal control. The fold changes of target genes expression relative to those of control group were analyzed by the 2^−ΔΔCT^ method [16], divided into different ranges and depicted as different colors.

### Effects of Hollow Gold Nanoshells on HAECs Tube Formation

To measure the effect of hollow gold nanoshells on angiogenesis of HAECs, tube formation assay was assessed by Matrigel basement membrane matrix [17].

In this study, 50 μL per well of Matrigel basement membrane matrix (Becton Dickinson, Bedford, MA, USA), which was used as extracellular matrix support, was added to a 96-well plate and allowed to gel for 60 min at 37 °C. Then, HAECs were seeded at a density of 1.5 × 10^4^ cells per well on the surface of the gel with or without 0.8 μg/mL hollow gold nanoshells and incubated for 14 h at 37 °C in a CO_2_ incubator. Meanwhile, the high urea solution (6 M urea) was used as a positive control for tube formation inhibition. The cultures on the gel were fixed for 10 min in 25 % glutaraldehyde, washed, and stained with Mayer’s hematoxylin. Each well was inspected under a light microscope at ×100 magnification and captured more than three pictures from different visual fields.

### Statistical Analysis

The data were represented as mean ± SD of more than four independent experiments. Statistical analysis was performed using one-way ANOVA followed by post hoc tests. A value of *p* < 0.05 was considered statistically significant.

## Results and Discussion

### Characterization of Hollow Gold Nanoshells

**Fig. 1:**
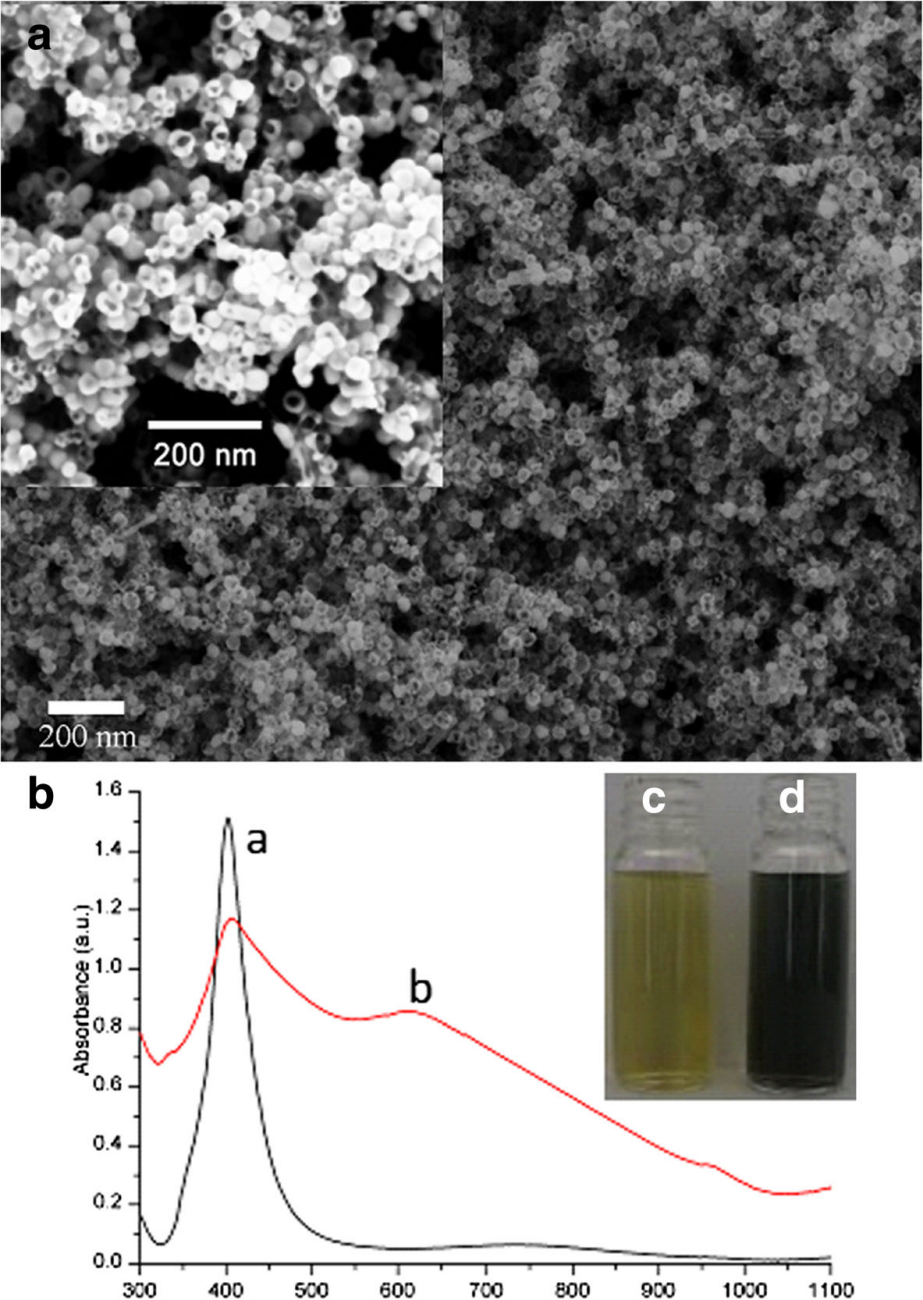
**a** The SEM image of as-obtained hollow gold nanoshells. *Inset* shows the image of these nanoshells at large magnification. **b** UV-vis spectra of (*a*) Ag nanoparticles solution (*b*) hollow gold nanoshells solution. Insets shows corresponding electronic pictures of (*c*) Ag nanoparticles solution and (*d*) hollow gold nanoshells solution

The morphology and structure of the hollow gold nanoshells were observed by SEM. Figure 1a shows the average diameter of hollow gold nanoshells is 35.6 ± 3.6 nm (*n* = 100). The narrow size distribution can avoid the difference of size effect. Figure 1b shows the comparison of UV-vis spectra of the obtained hollow gold nanoshells (b) and Ag nanoparticles (a) with sharp extinction at 391 nm and bright yellowish brown, which were used as sacrifice-template. The hollow gold nanoshells showed an average zeta potential of −38.9 mv, suggesting a good stability in suspension [18]. Moreover, the profile of the size distributions was highly sharp while symmetric (Additional file 1: Figure S1) and thus indicated that the hollow gold nanoshells were well dispersed in suspension.

### Internalization of Hollow Gold Nanoshells into HAECs

**Fig. 2:**
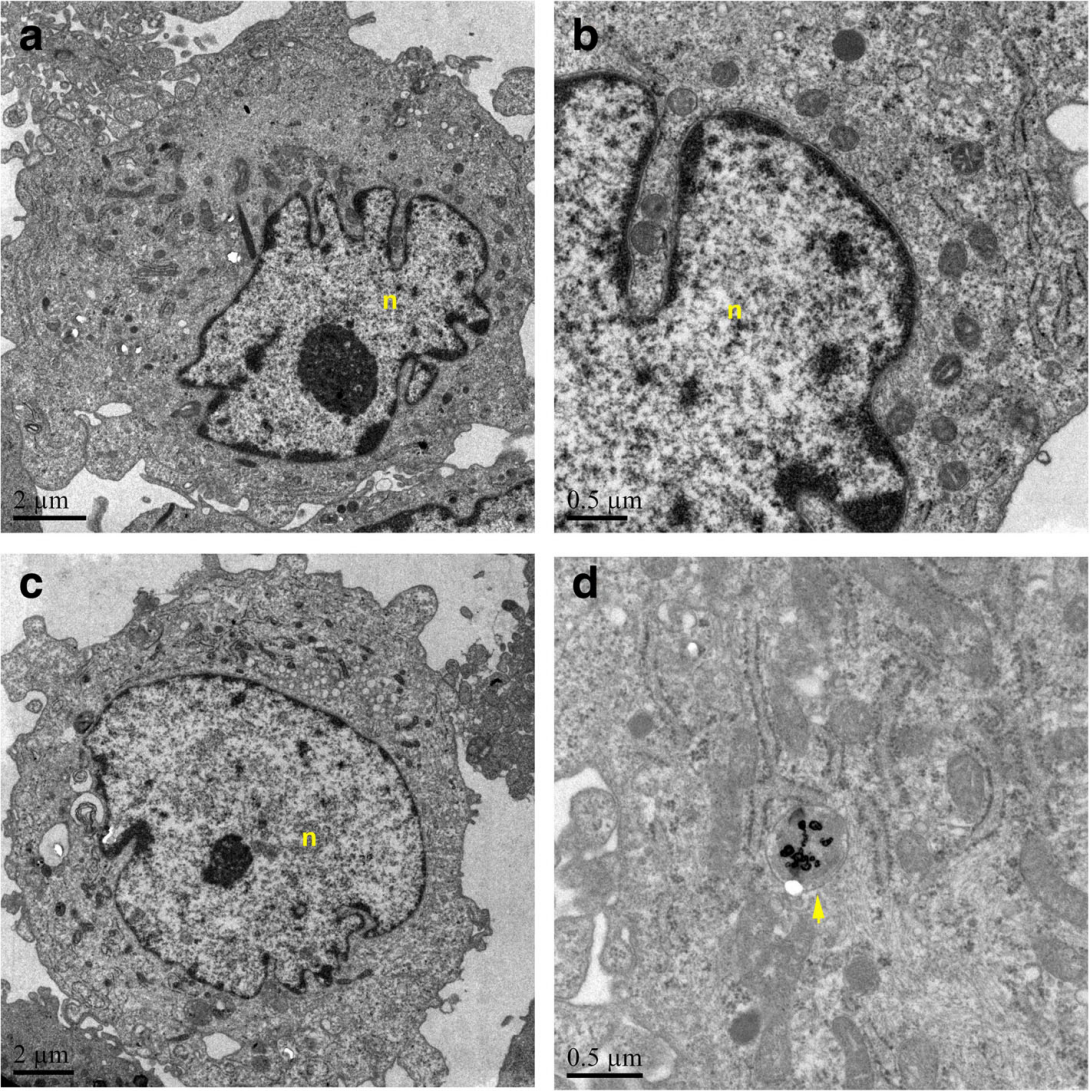
The TEM images of HAECs incubated with hollow gold nanoshells for 24 h. **a**, **b** HAEC without hollow gold nanoshells. **c**, **d** HAEC incubated with hollow gold nanoshells. The *scale bars* indicate 2 μm in **a** and **c** and 0.5 μm in **b** and **d**. *n* nucleus. *Arrows* denote the hollow gold nanoshells or particulate matter

The matter with high electron density in membrane-encircled cavities distinguished from the cellular structures was observed on TEM, which was consistent with the previous report [7]. Figure 2 represents TEM images between HAECs treated with 0.8 μg/mL hollow gold nanoshells (Fig. 2c, d) and HAECs without hollow gold nanoshell incubation (Fig. 2a, b). Moreover, no alterations of membrane integrity and mitochondrial injury were observed in HAECs treated with hollow gold nanoshells.

### Effects of Hollow Gold Nanoshells on HAECs Viability by MTT Assay

**Fig. 3:**
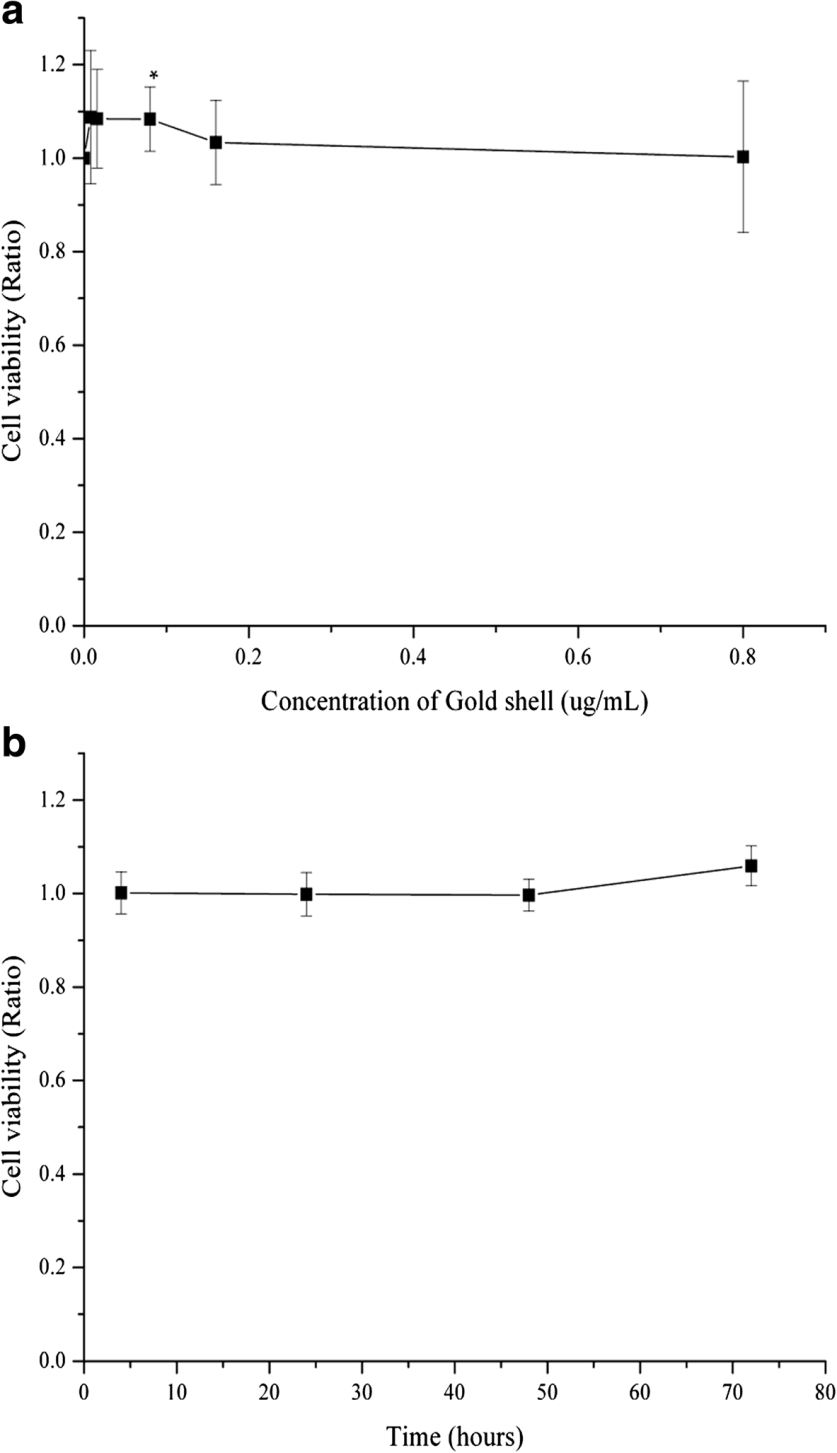
The cell viability of HAECs incubated with hollow gold nanoshells. Data are expressed as mean ± SD from independent experiments. Control values from HAECs incubated without hollow gold nanoshells were defined as 1. **a** HAECs were incubated with DMEM containing the gradient concentrations of hollow gold nanoshells for 24 h (0.008 to 0.8 μg/mL). **b** HAECs were incubated with DMEM containing 0.8 μg/mL hollow gold nanoshells for the indicated times (4, 24, 48, 72 h). *p < 0.05 vs. control

The cell viability of HAECs was 100.3 to 108.8 % when incubated with hollow gold nanoshells (0.008, 0.016, 0.08, 0.16, and 0.8 μg/mL) for 24 h compared with that in the control group (HAECs without hollow gold nanoshells), as shown in Fig. 3a. To observe the effect of duration of exposure, the cells were incubated with 0.8 μg/mL hollow gold nanoshells for 4, 24, 48, and 72 h, respectively (Fig. 3b). The data showed that there was no decreasing cell viability that occurred at any test time point and varied in a limited range from 99.6 to 105.9 % to control group. The results implied that there was not significant cytotoxicity effect of hollow gold nanoshells on HAECs, and the concentrations no more than 0.8 μg/mL were harmless.

### Effects of Hollow Gold Nanoshells on HAEC Injury Marker and Vasoregulators

**Fig. 4:**
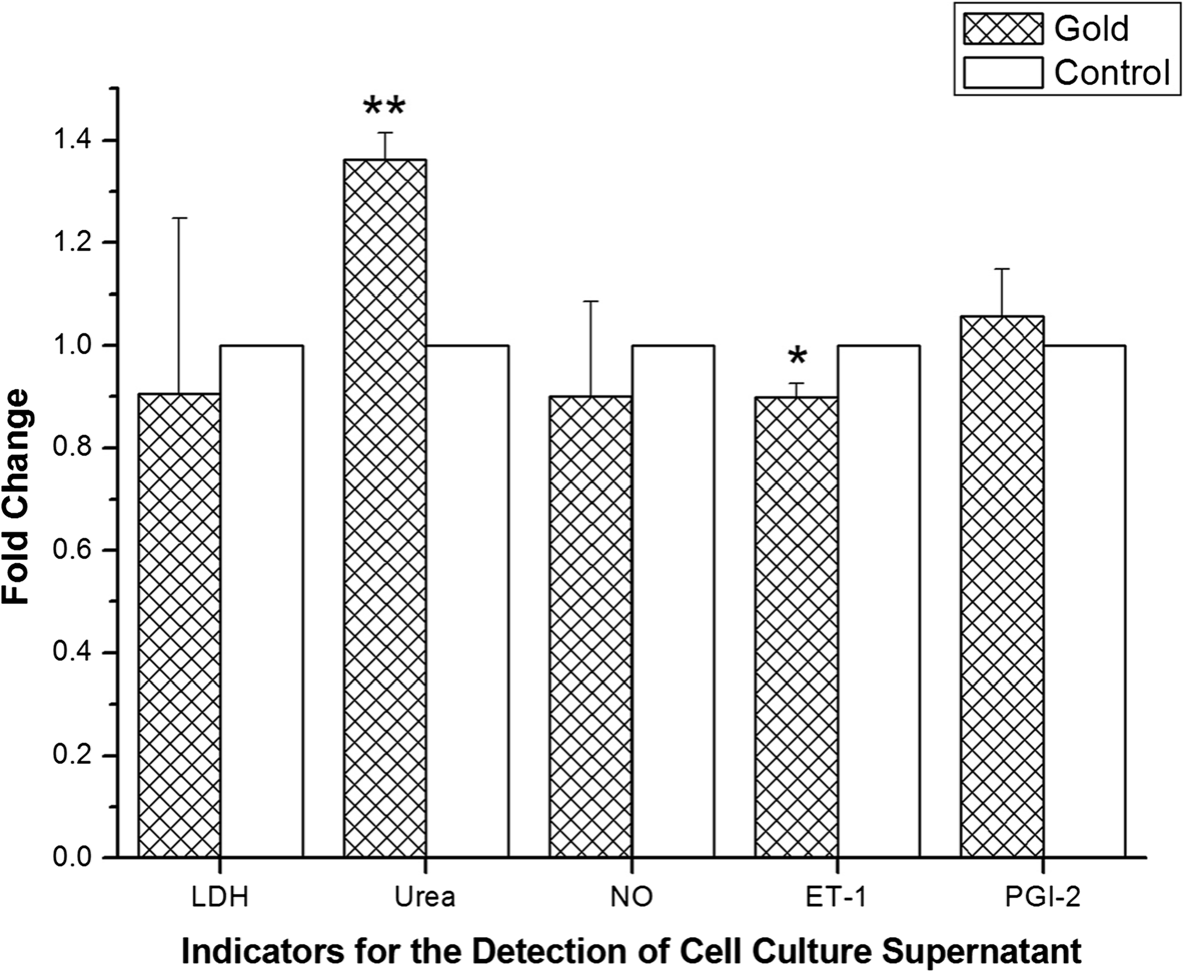
The levels of injury marker, LDH, and vasoregulators in supernatant. The HAECs were incubated with 0.8 μg/mL hollow gold nanoshells for 24 h. Ratios relative to control cells (without hollow gold nanoshells) are shown. *p < 0.05 vs. control; **p < 0.01 vs. control

The LDH released from HAECs incubated with 0.8 μg/mL hollow gold nanoshells for 24 h was not higher than that from control cells (Fig. 4), which was in accordance with the results of little cytotoxicity effect in MTT assay. As shown in Fig. 4, two of the important vasodilators released by ECs, NO and PGI-2, were not changed in hollow gold nanoshells treated HAECs. The vasoconstrictor ET-1 was significantly decreased in HAECs treated with 0.8 μg/mL hollow gold nanoshell for 24 h (Fig. 4, *p* < 0.05 vs. control group).

Taken together, the results implied that the balance between vasoconstrictor and vasodilator in HAECs, which can maintain normal tension of blood vessel, could be changed by hollow gold nanoshells. The suppression of vasoconstrictor ET-1 releasing from arterial ECs induces the dilation of blood vessel, which might be a benefit for treating some cardiovascular diseases, including hypertension [19] and atherosclerosis [20]. The mechanism for the reducing expression of ET-1 in HAECs by hollow gold nanoshells treatment needs to be further investigated.

In this study, the urea concentration in the HAECs treated with 0.8 μg/mL hollow gold nanoshell for 24 h was significantly higher than that in control cells (Fig. 4, *p* < 0.01), which suggested that the function of urea transporter in HAECs may be inhibited by hollow gold nanoshell exposure.

### Gene Expression on HAECs

**Fig. 5:**
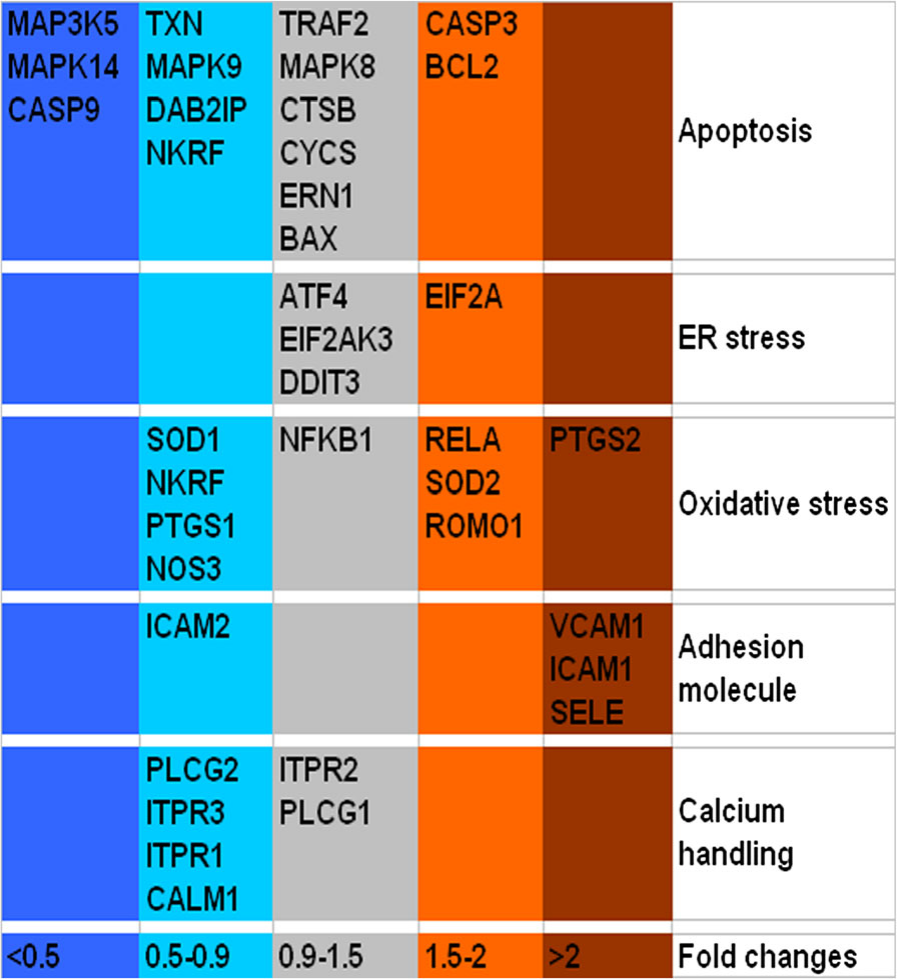
The fold changes in genes expression in HAECs incubated with hollow gold nanoshells. The results were analyzed by the 2^−ΔΔCT^ method. Gene symbols and corresponding encoded proteins: *MAP3K5* apoptosis signal-regulating kinase 1 (ASK1), *TRAF2* tumor necrosis factor receptor-associated factor 2 (TRAF2), *DAB2IP* ASK1-interacting protein (AIP1), *MAPK8* mitogen-activated protein kinase 8 (JNK1), *MAPK9* mitogen-activated protein kinase 9 (JNK2), *MAPK14* mitogen-activated protein kinase 14 (p38 MAPK α), *ERN1* endoplasmic reticulum to nucleus signaling 1 (IRE1), *BCL2* B cell lymphoma 2 (Bcl-2), *BAX* Bcl-2-associated X protein (Bax), *NKRF* nuclear factor Kb repressing factor, *TXN* thioredoxin, *CTSB* cathespin B, *CYCS* cytochrome C, *CASP9* caspase-9, *CASP3* caspase-3, *EIF2AK3* eukaryotic translation initiation factor 2α kinase 3 (PERK), *ATF4* activating transcription factor 4, *DDIT3* DNA-damage-inducible transcript 3 (CHOP), *EIF2A* eukaryotic translation initiation factor 2α, *NOS3* nitric oxide synthase 3 (eNOS), *SOD1* super oxide dismutase 1 (SOD-1), *SOD2* super oxide dismutase 2 (SOD-2), *ROMO1* reactive oxygen species modulator 1, *PTGS1* cyclooxygenase 1 (COX-1), *PTGS2* cyclooxygenase 2 (COX-2), *VCAM1* vascular cell adhesion molecule 1 (VCAM-1), *ICAM1* intercellular adhesion molecule 1 (ICAM-1), *ICAM2* intercellular adhesion molecule 2 (ICAM-2), *SELE* endothelial-leukocyte adhesion molecule 1 (E-selectin), *PLCG1* phospholipase C γ1, *PLCG2* phospholipase C γ2, *ITPR1* inositol 1,4,5-trisphosphate receptor type 1 (IP3R1), *ITPR2* inositol 1,4,5-trisphosphate receptor type 2 (IP3R2), *ITPR3* inositol 1,4,5-trisphosphate receptor type 3 (IP3R3), *CALM1* calmodulin 1 (CAM1)

The changes of gene expression related to apoptosis cascade, endoplasmic reticulum (ER) stress, oxidative stress, adhesion molecules, and calcium-handling proteins were depicted in Fig. 5. After HAECs incubated with 0.8 μg/mL hollow gold nanoshell for 24 h, most of the genes varied between 0.5- to 2-fold compared to those of HAECs without hollow gold nanoshell, except *MAPK14* (mitogen-activated protein kinase 14, MAPK14), *MAP3K5* (mitogen-activated protein kinase kinase kinase 5), *CASP9* (caspase 9), and *PTGS2* (prostaglandin-endoperoxide synthase 2). The expressions of *MAPK14*, *MAP3K5*, and *CASP9* in hollow gold nanoshells treated HAECs decreased to less than 0.5-fold to those of the normal control cells, which implied that the genes involved in apoptosis cascade were not activated in this study. On the contrary, the expression of *PTGS2*, which encodes cyclooxygenase-2 (COX-2), increased to above twofold in hollow gold nanoshell-treated HAECs. COX-2 is a key enzyme in the synthesis of prostaglandins and thromboxanes [21] and is induced by inflammatory stimuli such as bacterial endotoxin and cytokines [22]. Endothelial cell COX-2 promotes endothelial cell adhesion, spreading, migration and angiogenesis through the prostaglandin-cAMP-PKA-dependent pathway [23]. Previous research reported that the expression of COX-2 could not be enhanced by gold nanoparticles of varied sizes in RAW 264.7 mouse macrophages [24]. But a different result was reported on gold nanorod [25]. The disparity may be related to the shape, size, concentration, and duration of exposure of the gold nanoparticles. In this study, the hollow gold nanoshells increased the expression of inducible COX-2 in HAECs. It was noted that *PTGS1*, which encodes constitutive COX-1 isoform, could not be increased by hollow gold nanoshells treatment in HAECs.

Up-regulation also occurred in the adhesion molecular genes *ICAM1* (intercellular adhesion molecule 1, ICAM-1), *VCAM1* (vascular cell adhesion protein 1, VCAM-1) and *SELE* (endothelial-leukocyte adhesion molecule 1, E-selectin), as shown in Fig. 5. Whereas, the expression of *ICAM-2* was slightly decreased by hollow gold nanoshells treatment. ICAM-1 and -2 belong to integrin-binding Ig superfamily adhesion molecules that are important for leukocyte transmigration across endothelial monolayers. ICAM-1 is expressed at very low levels whereas ICAM-2 is expressed abundantly in the membranes of resting endothelial cells [26, 27]. The ICAM-1 level was increased by proinflammatory cytokines, and the ICAM-2 level was concomitantly decreased. In endothelial cells, ICAM-1, but not ICAM-2, rapidly stimulates signaling responses involving RhoA and alters actin cytoskeletal organization [26]. VCAM-1 and E-selectin are cell adhesion molecules expressed only after the endothelial cells are stimulated by cytokines and thus play an important part in inflammation. In this study, the increasing expressions of ICAM-1, VCAM-1, and E-selection suggested that hollow gold nanoshells evoked similar effects to inflammatory stimuli or cytokines in HAECs.

### Effects of Hollow Gold Nanoshells on HAECs Tube Formation

**Fig. 6:**
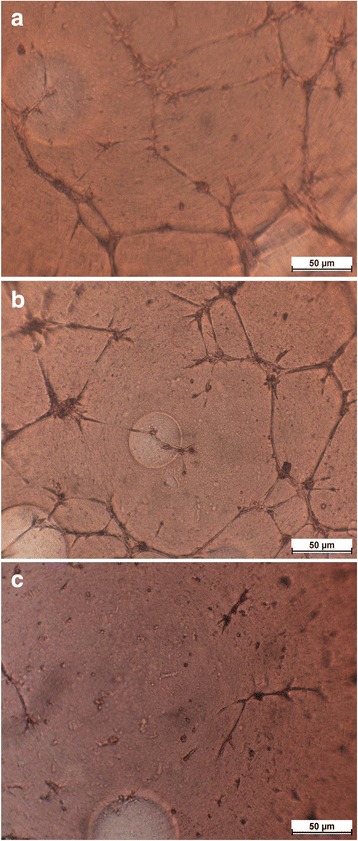
Effect of hollow gold nanoshells on tube network formed by HAECs cultured on Matrigel within 14 h. **a** HAECs can form a capillary-like network on Matrigel-coated wells within 14 h. **b** No obvious change to form networks by HAECs in the presence of 0.8 μg/mL hollow gold nanoshells. **c** The high urea solution (6 M urea) was used as a positive control for inhibition of tube formation

Angiogenesis is the formation of new capillaries from preexisting blood vessels. This process involves the migration, growth, and differentiation of endothelial cells and may be induced by kinds of mediators including cytokines, growth factors, and cell adhesion molecules. Angiogenesis occurs in the process of development and wound healing physiological conditions. However, pathological angiogenesis plays an essential role in tumor, retinopathy, and rheumatoid arthritis. Therefore, the boost and inhibition of angiogenesis have been the focus of basic and clinical research. The tube formation assay is a convenient method to study biochemical and molecular events associated with angiogenesis in vitro. HAECs of control group (without hollow gold nanoshells treatment, Fig. 6a) can form a capillary-like network on Matrigel-coated wells within 14 h. On the opposite, HAECs treated with 6 M urea (Fig. 6c) failed to form tubes due to its high osmolality. Hollow gold nanoshell treatment could not decrease the tubes formation of HAECs in this study (Fig. 6b). The result of tube formation assay suggested that the hollow gold nanoshells had no effect on inhibiting angiogenesis of ECs.

Previous research [28] reported that nanogold bound to heparin-binding growth factors like vascular endothelial growth factor 165 (VEGF165) and basic fibroblast growth factor (bFGF) and inhibit their activity, whereas it did not inhibit the activity of non-heparin-binding growth factors like VEGF121 and endothelial growth factor (EGF). The VEGF-induced angiogenesis was inhibited by gold nanoparticles via inhibiting Akt phosphorylation [29, 30] and blocking VEGFR2 auto-phosphorylation to inhibit consequently extracellular signal-related kinase 1/2 (ERK 1/2) activation [31]. Gold nanoparticles coated with different functional peptide are demonstrated to activate or inhibit in vitro angiogenesis [32]. In this study, hollow gold nanoshells presented a different result on angiogenesis with other nanogolds, which needs to be further investigated.

## Conclusions

In this study, hollow gold nanoshells with a 35-nm diameter and a 10-nm shell thickness were synthesized based on galvanic replacement using silver nanoparticles as sacrifice-template. The naked hollow gold nanoshells can be internalized into the HAECs without disrupting the cellular morphology and membrane integrity. The secretion of vasoconstrictor ET-1 was decreased in HAECs treated with hollow gold nanoshells. Hollow gold nanoshells could suppress the expressions of genes related to apoptosis and activate the expression of certain adhesion molecules of HAECs. The angiogenesis by the HAECs were not inhibited. Altogether, this study provides a new shape of nanogold without impairing the structure and major function of normal arterial endothelial cells, which might be a potential tool for drug carrier.

## Additional file

Additional file 1: Figure S1. A representative profile of size distributions of the hollow gold nanoshells in suspension. (DOCX 42 kb)

## Abbreviations

bFGF: Basic fibroblast growth factor
DMEM: Dulbecco’s modified Eagle’s medium
DMSO: Dimethyl sulfoxide
EGF: Endothelial growth factor
ELISA: Enzyme-linked immunosorbent assay
ER: Endoplasmic reticulum
ERK: Extracellular signal-related kinase
ET-1: Endothelin-1
HAEC: Human aortic endothelial cells
HUVEC: Human umbilical vein endothelial cells
LDH: Lactate dehydrogenase
MTT: 3-(4,5-Dimethyl-2-thiazolyl)-2,5-diphenyl-2-H-tetrazolium bromide
NO: Nitric oxide
PBS: Phosphate buffer solution
PGI-2: Prostacyclin I-2
SEM: Scanning electron microscopy
TEM: Transmission electron microscopy
VEGF: Vascular endothelial growth factor
