# Supplementary figures and images for: In Vitro Effects of Hollow Gold Nanoshells on Human Aortic Endothelial Cells
Source: Nanoscale Res Lett. 2016 Sep 13;11(1):397. doi: 10.1186/s11671-016-1620-5 (PMC5021651; doi:10.1186/s11671-016-1620-5)

**
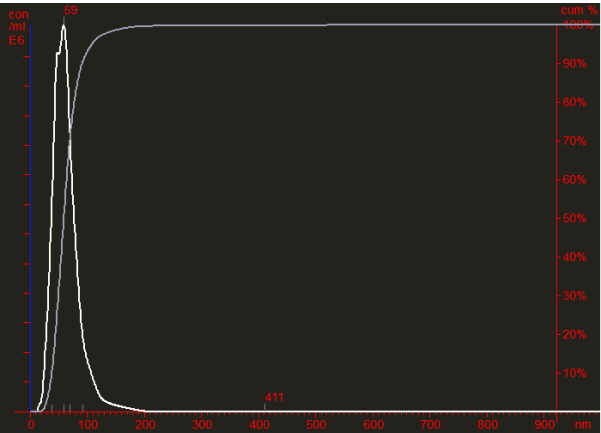
**

Supplement: Additional file 1: Figure S1. — A representative profile of size distributions of the hollow gold nanoshells in suspension. (DOCX 42 kb) [file 11671_2016_1620_MOESM1_ESM.docx]
